# Resting‐State Brain Entropy Reveals Altered Neural Dynamics and Cognitive Associations in Mild Traumatic Brain Injury: A Longitudinal Study

**DOI:** 10.1002/jnr.70153

**Published:** 2026-08-28

**Authors:** Li Jiang, Ze Wang, Steven Roys, Rosy Linda Njonkou Tchoquessi, Prashant Raghavan, Neeraj Badjatia, Rao P. Gullapalli, Jiachen Zhuo

**Affiliations:** ^1^ Department of Diagnostic Radiology and Nuclear Medicine University of Maryland School of Medicine Baltimore Maryland USA; ^2^ Center for Advanced Imaging Research (CAIR) University of Maryland School of Medicine Baltimore Maryland USA; ^3^ Department of Psychiatry University of Maryland School of Medicine Baltimore Maryland USA; ^4^ Department of Otorhinolaryngology‐Head & Neck Surgery University of Maryland School of Medicine Baltimore Maryland USA; ^5^ Department of Neurology University of Maryland School of Medicine Baltimore Maryland USA; ^6^ Department of Neurosurgery University of Maryland School of Medicine Baltimore Maryland USA

**Keywords:** brain entropy, cognitive recovery, longitudinal study, mild traumatic brain injury, resting‐state fMRI

## Abstract

Mild traumatic brain injury (mTBI) is associated with persistent physical, cognitive, and emotional symptoms, yet the functional brain changes that accompany recovery remain incompletely understood. This longitudinal study investigated brain entropy (BEN), a resting‐state measure of irregularity or complexity of spontaneous BOLD activity derived from resting‐state functional magnetic resonance imaging (rs‐fMRI), during the first year following mTBI. Data of rs‐fMRI were acquired from 48 patients with mTBI at the acute (< 14 days), 6‐month, and 12‐month post‐injury visits, and 34 healthy controls (HCs). BEN was quantified using sample entropy. Patients with mTBI also underwent behavioral assessments evaluating quality of life and cognitive function. Behavioral assessments demonstrated progressive improvements in patient‐reported quality of life and objective cognitive performance. Cross‐sectional analyses revealed dynamic, time‐dependent alterations in BEN. Compared with HCs, patients with mTBI exhibited higher BEN in precuneus, temporal, occipital, and sensorimotor regions during the acute and 6‐month stages, whereas lower BEN was observed in sensorimotor, occipital, and frontoparietal regions at the 12‐month follow‐up. Longitudinal analyses demonstrated progressive regional reductions in BEN over time. Several associations between longitudinal changes in regional BEN and cognitive performance were nominally significant, particularly in the precuneus, but none survived multiple comparison corrections. These findings indicate that mTBI recovery is accompanied by dynamic, regionally specific changes in resting state BOLD irregularity. BEN may therefore provide a promising imaging biomarker for tracking the evolving neural dynamics underlying recovery following mTBI.

## Significance Statement

Although functional brain alterations following mild traumatic brain injury (mTBI) have been widely reported, their longitudinal evolution remains poorly understood. This study demonstrates dynamic changes in brain entropy across the first year after injury, together with progressive recovery in cognitive performance and patient‐reported quality of life. By integrating longitudinal neuroimaging and behavioral assessments, the findings provide new evidence that brain entropy captures evolving neural dynamics during recovery and may serve as a promising noninvasive imaging biomarker for monitoring recovery following mTBI.

## Graphical abstract

Resting‐state brain entropy mapping in mild TBI revealed dynamic, regional‐specific changes from acute to chronic stages. Early hyperentropy in precuneus, a key hub of DMN, predicted short‐term cognitive gains, while later shifts and entropy reductions aligned with long‐term recovery, highlighting evolving functional changes after injury.

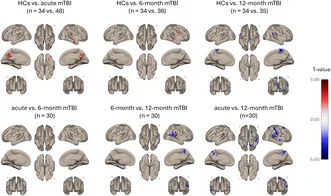

## 1 Introduction

Mild traumatic brain injury (mTBI), accounting for approximately 70%–90% of all traumatic brain injuries, is a significant public health issue affecting millions of individuals worldwide (Cassidy et al. 2014; Donovan et al. 2014; Mott et al. 2012). Although termed “mild,” mTBI can lead to persistent physical, cognitive, and emotional impairments in up to 30% of affected individuals, often presenting as post‐concussive symptoms that interfere with daily functioning (Cancelliere et al. 2023; Sheldrake et al. 2022; McInnes et al. 2017). A major challenge is understanding how these subtle injuries disrupt the intrinsic organization and information processing of large‐scale neural networks.

Resting‐state functional magnetic resonance imaging (rs‐fMRI) has advanced our understanding of the brain's functional architecture following mTBI, revealing widespread alterations in connectivity and network topology (Sours et al. 2013, 2015). Disruptions have been consistently observed in large‐scale networks such as the default mode, executive control, and salience networks (Sharp et al. 2011; Vedaei et al. 2021; Dogra et al. 2024; Zhan et al. 2015). While informative, these findings do not fully capture the multifaceted functional brain alterations associated with mTBI.

Brain entropy (BEN), derived from the rs‐fMRI signals, quantifies the irregularity or complexity of spontaneous neural activity. It is increasingly recognized as a marker of intrinsic neural dynamics, reflecting the brain's capacity for flexible information processing and dynamic state transitions. Higher entropy values have been associated with greater variability and adaptability in brain function, while lower entropy may reflect reduced neural complexity or increased rigidity in network behavior. This relationship between entropy and neural dynamics has been supported by theoretical and empirical evidence linking entropy to functional integration and segregation, cognitive performance, and age‐ or disease‐related alterations in brain function (Wang et al. 2014; Wang 2021; Song and Wang 2025a, 2025b; Camargo et al. 2024; Del Mauro et al. 2026; Donghui Song et al. 2019; Lin et al. 2022). In clinical contexts, altered BEN has been observed in various conditions, including aging, Alzheimer's disease, depression, schizophrenia, and neurodegenerative diseases, often interpreted as reflecting disrupted neural dynamics and diminished cognitive capacity (Del Mauro, Li, et al. 2025; Del Mauro et al. 2024; Del Mauro and Wang 2024; Del Mauro, Zeng, and Wang 2025; Drachman 2006; Liu et al. 2020; Wang and Alzheimer's Disease Neuroimaging Initiative 2020; Xue et al. 2019; Yin et al. 2023; Zhou et al. 2016; Li, Fang, et al. 2016; Wang et al. 2017; Jiang et al. 2023). Accordingly, evaluating BEN following mild TBI may provide insights into the evolving pathophysiology of brain dysfunction and its relationship with cognitive outcomes across acute and chronic phases of injury. Furthermore, previous studies have demonstrated that regional BEN can be modulated noninvasively in both healthy individuals and clinical populations, suggesting its potential utility as a therapeutic target (Jordan et al. 2024; Liu et al. 2025; Song et al. 2025, 2019; Chang et al. 2018). However, its application in mTBI remains relatively underexplored.

Because substantial functional recovery following mTBI is known to occur within the first year after injury, this period represents a critical window for investigating the neural mechanisms underlying recovery (Nelson et al. 2023; McCrea et al. 2021; McGeown et al. 2026). In this longitudinal study, we employed BEN analysis to investigate longitudinal changes in brain function throughout this clinically important recovery period. We hypothesized that patients with mTBI would exhibit dynamic changes in BEN during the first year after injury, accompanied by improvements in cognitive performance. Specifically, our objectives were to: (1) quantify and compare BEN between healthy controls (HCs) and patients with mTBI at three clinically relevant time points (acute (< 14 days), 6 months, and 12 months post‐injury); (2) characterize longitudinal changes in BEN within the mTBI group over the first year after injury; and (3) examine the relationship between longitudinal changes in BEN within significant brain regions and cognitive performance in patients with mTBI. By addressing these objectives, we sought to improve our understanding of the neural mechanisms underlying recovery following mTBI and to evaluate the potential of BEN as an imaging biomarker for monitoring recovery.

## 2 Methods

### 2.1 Participants

The study was approved by the Institutional Review Board of the University of Maryland, Baltimore, and written informed consent was obtained from all participants. Participant recruitment procedures have been described previously (Zhuo et al. 2024) and the key eligibility criteria are summarized below. Patients with mTBI were recruited from the R Adams Cowley Shock Trauma Center at the University of Maryland Medical Center, whereas HCs were recruited from the local community. All participants were between 18 and 60 years of age, English‐speaking, and had no contraindications to MRI.

Patients with mTBI were eligible if they were admitted to the trauma center within 24 h of injury, had a Glasgow Coma Scale (GCS) score of 13–15, and met at least one of the following criteria: (1) positive head CT findings or (2) negative head CT findings accompanied by documented or reported loss of consciousness, post‐traumatic amnesia, altered mental status, and/or evidence of facial trauma. Exclusion criteria included penetrating head injury, previous TBI requiring medical attention within the past five years or any prior TBI resulting in persistent neurological or psychological deficits, major neurological or psychiatric disorders requiring active treatment, pregnancy, and inability to comply with the longitudinal follow‐up protocol. Post‐traumatic stress disorder (PTSD) was not an exclusion criterion. HCs were required to have no history of TBI or major neurological or psychiatric disorders.

All patients with mTBI underwent MRI and a battery of behavioral assessments, including the TBI Quality of Life questionnaire (TBI‐QoL), NIH Toolbox cognitive assessments, and the PTSD Checklist for DSM‐5 (PCL‐5), at three post‐injury time points: acute (< 14 days), approximately 6 months, and approximately 12 months after injury. Due to incomplete follow‐up, the final dataset included 48 participants at the acute visit, 36 at the 6‐month visit, and 35 at the 12‐month visit. Longitudinal BEN analyses were performed on a subset of 30 participants who completed rs‐fMRI at all three time points.

Twenty‐one HCs underwent MRI, TBI‐QoL questionnaire, and NIH Toolbox cognitive assessments at a single study visit. To increase the sample size for the cross‐sectional BEN analysis, an additional 11 healthy participants from other research projects were included. These participants were scanned using the same MRI scanner and identical rs‐fMRI acquisition protocol, and their imaging data were processed using the same processing pipeline. Behavioral questionnaires and NIH Toolbox cognitive assessments were not available for these additional participants. Consequently, a total of 34 HCs were included in the BEN analyses, whereas quality‐of‐life and cognitive analyses were restricted to the original cohort of 21 HCs.

### 2.2 Behavioral Assessment

#### 2.2.1 TBI Quality of Life Questionnaire

The TBI‐Quality of Life (TBI‐QoL) questionnaire comprises 17 items distributed across four main domains: Cognition, Emotional Health, Physical Health, and Social Participation (Tulsky and Kisala 2019; Tyner et al. 2020). Domain scores are calculated by summing the items within each respective domain, as detailed in Table S1. Notably, the directionality of item scoring varies for most items (e.g., within Cognition and Social Participation), higher scores indicate better function or well‐being. However, within the Emotional Health and Physical Health domains, specific items are designed to measure symptom severity (e.g., Depression, Fatigue), where higher scores reflect greater burden. This framework provides a comprehensive, multi‐dimensional assessment of quality of life in TBI patients, capturing both functional strengths and symptom burden.

#### 2.2.2 NIH Toolbox Cognition Battery

The NIH toolbox cognition battery (NIHTB‐CB) (Gershon et al. 2013; Weintraub et al. 2014) was utilized to assess participants' cognitive function, measuring both crystallized and fluid cognitive abilities. As detailed in Table S2, crystallized cognition was assessed by the picture vocabulary test (language and vocabulary knowledge). Scores from this test were used to form a Crystallized Cognition Composite Score, also known as the early childhood composite Score, which is largely resistant to TBI effects (Tulsky et al. 2017) and can be used for an estimation of pre‐injury general cognitive ability. The fluid cognition tests included the list sorting working memory test, picture sequence memory test, auditory verbal learning test (rey), pattern comparison processing speed test, flanker inhibitory control and attention test, and dimensional change card sort test. Scores from these six tests were combined into a fluid cognition composite score, which is sensitive to TBI severity (Tulsky et al. 2017; Holdnack et al. 2017). In this study, age‐adjusted scale scores were employed to assess group differences and track longitudinal cognitive changes.

### 2.3 MRI Data Acquisition

All the MRI scans were conducted on the same 3 T Siemens Prisma scanner using a 64‐channel head and neck coil. Data of rs‐fMRI were acquired with a 2D multi‐band echo‐planar imaging (EPI) sequence with the following parameters: TR = 943 ms, TE = 30 ms, slice‐acceleration factor = 4, flip angle = 65°, averages = 1, field of view (FoV) = 240 × 240 mm^2^, resolution = 3 × 3 × 3 mm^3^, and matrix size = 80 × 80 × 56. A total of 500 brain volumes were collected over an acquisition time of 8 min. Foam padding was used to restrain head motion during scanning. Participants were instructed to remain awake with their eyes open during rs‐fMRI acquisitions.

High‐resolution 3D isotropic T1‐weighted MPRAGE images were acquired in the sagittal plane with the following parameters: TR = 4000 ms, TE = 3.37 ms, inversion time (TI) = 1400 ms, averages = 1, flip angle = 6°, FoV = 256 × 256 mm^2^, GRAPPA acceleration (phase‐encoding direction) = 2, acceleration factor (3D) = 1, resolution = 1 × 1 × 1 mm^3^, and matrix size = 192 × 256 × 256. The total acquisition time was 8 min and 14 s.

### 2.4 MRI Data Processing and BEN Mapping

#### 2.4.1 Preprocessing and rs‐fMRI Data Denoising

The structural MRI and resting‐state fMRI (rs‐fMRI) data were preprocessed using the CONN toolbox (version 22a) (http://www.nitrc.org/projects/conn, RRID: SCR_009550). The preprocessing steps for the structural T1‐MPRAGE images involved normalization to the MNI152 template space with a spatial resolution of 1 × 1 × 1 mm^3^ and simultaneous segmentation into gray matter (GM), white matter (WM), and cerebral spinal fluid (CSF) using the prior tissue probability distribution map (Ashburner and Friston 2005).

For the rs‐fMRI data, the preprocessing steps included motion estimation and correction through realignment and unwarp, slice timing correction, artifact detection using the ART method, simultaneous segmentation of GM/WM/CSF, normalization to the standard MNI template with a resolution of 2 × 2 × 2 mm^3^, and spatial smoothing with a 6 mm full‐width at half‐maximum (FWHM) Gaussian kernel. During the artifact detection preprocessing step, quality control (QC) timeseries were computed, including Global Signal Change (GSC) and Framewise Displacement (FD). Quality control was subsequently performed as described in (Morfini et al. 2023). Data of rs‐fMRI with excessive head motion, defined as a maximum FD > 0.5 mm or more than 30% of volumes identified as motion outliers, were excluded from subsequent statistical analysis.

Further denoising of the preprocessed rs‐fMRI data included nuisance regression, temporal band‐pass filtering (0.01–0.1 Hz), and linear detrending to reduce non‐neuronal noise and residual head motion effects in the BOLD signal. Nuisance regressors included the first five principal components of the white matter (WM) and cerebrospinal fluid (CSF) signals (aCompCor), the Friston 24‐parameter motion model (six rigid‐body realignment parameters, their first‐order temporal derivatives, and the squared terms of both), and scrubbing regressors for motion‐contaminated volumes (Morfini et al. 2023).

#### 2.4.2 BEN Mapping

BEN maps were calculated using the BEN Mapping Toolbox (BENtbx) for MATLAB (The MathWorks Inc., Natick, MA, USA) (Wang et al. 2014). BENtbx calculates Sample Entropy (SampEn), a widely used measure for quantifying the predictability and complexity of time‐series data, particularly stable for fMRI time‐series analysis. Preprocessed and denoised rs‐fMRI data within the FSL MNI152 template brain mask were used as input for BEN map calculation.

For each voxel, the BOLD time series was divided into overlapping segments of length 𝑚. The Chebyshev distance between all pairs of segments was computed, and if the distance was less than a tolerance threshold r, defined as r×stdof the time series, the segments were considered a match. This process iterated across all segments, first for m and then for m+1 to calculate the probability of matches, $B_{m}\left(r\right)$ and $A_{m+1}\left(r\right)$, respectively. SampEn was defined as the negative natural logarithm of the ratio of the two probabilities:

$$\text{SampEn}=-\ln\left(\frac{A_{m+1}\left(r\right)}{B_{m}\left(r\right)}\right)$$

**FIGURE 1:**
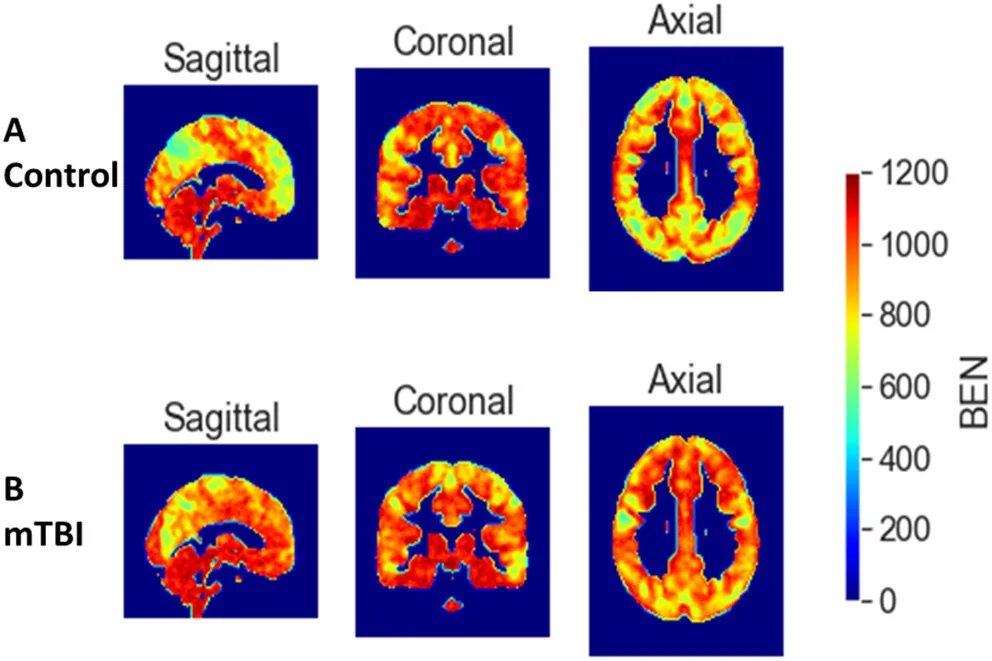
Representative brain entropy maps from a healthy control (HC) and a patient with mild traumatic brain injury (mTBI), displayed in three orthogonal views (axial, sagittal, and coronal).

As suggested by the BENtbx, the two parameters were set to m=3 and r=0.6. Entropy values were computed voxel‐wise, resulting in 3D BEN maps for each rs‐fMRI dataset. Representative BEN maps for both HC and mTBI participants are illustrated in Figure 1.

### 2.5 Statistical Analysis

#### 2.5.1 Demographics and Clinical Data

For demographic characteristics, continuous variables were compared between groups using Welch's *t*‐test to account for unequal variances, whereas categorical variables were compared using the chi‐square (*χ* ^2^) test or Fisher's exact test, as appropriate. Clinical characteristics of the mTBI group were summarized descriptively as mean ± standard deviation (SD) or number (percentage).

#### 2.5.2 Behavioral Assessments

Behavioral assessment data consisted of scores derived from patient‐reported outcome of TBI‐QoL and the NIH Toolbox Cognition Battery. To robustly compare these behavioral assessment scores between HCs and patients with mTBI across three longitudinal time points (acute, 6‐month, and 12‐month post‐injury), we employed linear mixed models (LMMs). For each behavioral outcome, the LMM included group (HC vs. mTBI), time point, and their interaction (group × time) as fixed effects. Subject ID was modeled as a random intercept to account for the inherent within‐subject correlations due to repeated measures. To control for potential demographic confounding, age and sex were included as covariates for the TBI‐QoL measures. However, for the NIH Toolbox Cognition Battery scores, which were already age‐corrected, only sex was included as a covariate. This modeling approach efficiently handles missing data under the Missing At Random (MAR) assumption and provides statistically robust estimates for both group differences and longitudinal changes. To control the increased risk of Type I errors due to multiple comparisons across the various behavioral measures, False Discovery Rate (FDR) adjustment was applied, setting the adjusted significance threshold at *p* < 0.05 (FDR‐adjusted). All statistical analyses were conducted using Python statsmodels (v0.14.5) for LMM implementation and FDR correction.

#### 2.5.3 Cross‐Sectional and Longitudinal BEN Analysis

Voxel‐wise statistical analyses were performed within the general linear model (GLM) framework implemented in SPM12. For the cross‐sectional analyses, group comparisons between HCs and patients with mTBI were conducted separately using the acute (*n* = 48), 6‐month (*n* = 36), and 12‐month (*n* = 35) post‐injury mTBI cohorts. Voxel‐wise two‐sample *t*‐tests with age and sex included as covariates were conducted to evaluate the group differences.

For the longitudinal analyses, only patients with mTBI who completed MRI scans at all three post‐injury time points (*n* = 30) were included. HCs were not included because only a single MRI scan was available for each participant. Longitudinal changes in brain entropy were assessed using voxel‐wise pairwise *t*‐test between the acute and 6‐month, acute and 12‐month, and 6‐month and 12‐month post‐injury time points.

For both cross‐sectional and longitudinal analyses, statistical inference was performed using a cluster‐defining threshold of voxel‐wise uncorrected *p* < 0.005, followed by cluster‐level FDR correction at *p* < 0.05. Significant clusters were visualized using the CONN toolbox display utility, and anatomical labels were assigned according to the Harvard–Oxford cortical and subcortical atlases implemented in CONN.

#### 2.5.4 Correlation Between Entropy Change and Cognitive Change Within mTBI Group

To investigate the relationship between longitudinal changes in BEN and cognitive function, partial Pearson correlation analyses were performed in the subset of 30 patients with mTBI who had completed MRI data at all three study visits. Significant clusters identified from the longitudinal voxel‐wise analyses were anatomically labeled using the CONN toolbox and used to define regions of interest (ROIs). Mean BEN was extracted from each ROI at each visit, and longitudinal changes in entropy and NIH Toolbox cognitive scores were calculated over the same recovery intervals (6 months–acute, 12 months–6 months, and 12 months–acute). Partial Pearson correlation analyses, controlling for age and sex, were performed to assess the associations between changes in regional BEN and cognitive performance. To account for multiple comparisons, *p*‐values were adjusted using the Benjamini–Hochberg FDR procedure, with statistical significance defined as an FDR corrected *q* < 0.05.

## 3 Results

### 3.1 Demographic and Clinical Results

Demographic and clinical characteristics of the participants are summarized in Table 1. A total of 48 patients with mTBI and 34 HCs were included in this study. There were no significant differences in age between the two groups (34.79 ± 9.90 vs. 36.50 ± 14.13 years, *p* = 0.565). However, the groups differed significantly in sex distribution (*p* = 0.018) and years of education (*p* = 0.003), with the mTBI group having a higher proportion of males and fewer years of education than the HC group. Race distribution also differed significantly between the groups (*p* = 0.034), while ethnicity did not differ significantly (*p* = 1.000).

**TABLE 1:** Demographics and clinical characteristics of HCs and mTBI participants at enrollment.

Categories	mTBI (*n* = 48)	HC (*n* = 34)	*p*
*Demographics*			
Age, years	34.79 ± 9.90	36.5 ± 14.13	0.565
Sex, male/female	28/20	10/24	**0.018**
Education, years	14.10 ± 2.12	16.72 ± 3.31^a^	**0.003**
Race			**0.034**
White	21 (43.8%)	16 (47.1%)	
Black/African American	20 (41.7%)	10 (29.4%)	
Asian	1 (2.1%)	6 (17.7%)	
Other	6 (12.5%)	2 (5.9%)	
Ethnicity			1.000
Non‐hispanic	41 (85.4%)	25 (92.6%)	
Hispanic	7 (14.6%)	2 (7.41)	
*Clinical characteristics*			
Admission GCS	14.69 ± 0.60	─	
Loss of consciousness	27 (56.3%)	─	
Alternation of consciousness	18 (37.5%)	─	
Facial trauma	34 (70.8%)	─	
Amnesia	8 (16.7%)	─	
Positive head CT/MRI	16 (33.3%)	─	
Positive post‐traumatic stress symptoms^b^	7 (14.6%)	─	

*Note:* Data are presented as mean ± SD or *n* (%). Abbreviations: CT, computed tomography; GCS, Glasgow Coma Scale; HC, healthy control; MRI, magnetic resonance imaging; mTBI, mild traumatic brain injury.

^a^ Education data were unavailable for 12 HCs.

^b^ Positive post‐traumatic stress symptoms were defined as a PTSD Checklist for DSM‐5 (PCL‐5) total score > 33.

Among patients with mTBI, the mean admission GCS was 14.69 ± 0.60, confirming that the cohort was predominantly comprised of patients with mild traumatic brain injury. More than half of the patients experienced loss of consciousness (56.3%) or alternation of consciousness (70.1%), and 16.7% reported post‐traumatic amnesia. Facial trauma was presented in 70.8% of patients, whereas positive findings on clinical CT or MRI were observed in 33.3% of patients. In addition, only 14.6% of patients with mTBI screened positive for post‐traumatic stress symptoms, defined as PCL‐5 total score greater than 30. Motor vehicle collision was the most common mechanism of injury (38.3%).

### 3.2 Behavioral Assessments

Unless otherwise specified, all reported *p*‐values for the behavioral analyses are corrected for multiple comparisons using the Benjamini‐Hochberg false discovery rate (FDR) procedure.

#### 3.2.1 TBI QoL Test Scores

**FIGURE 2:**
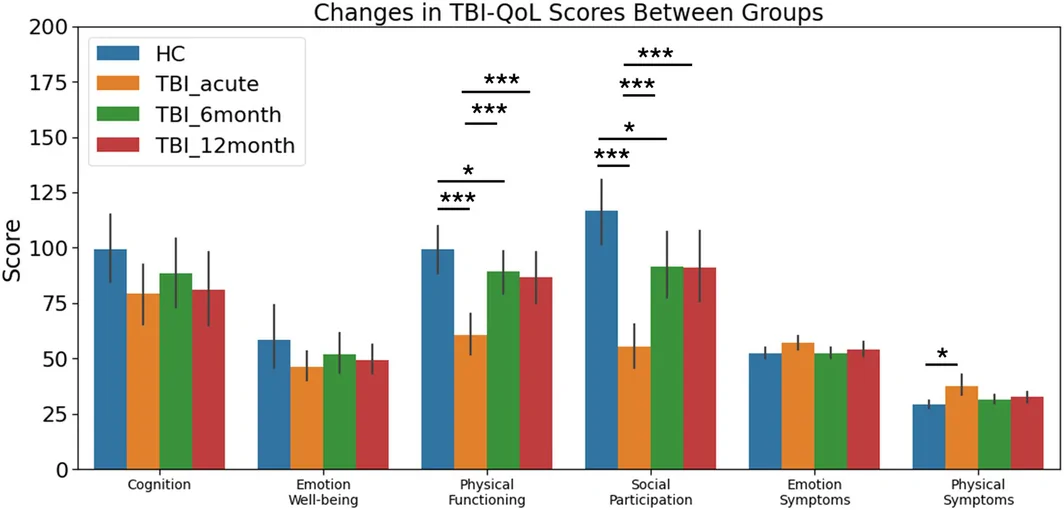
Linear mixed model (LMM) analysis results of TBI‐QoL composite scores by comparing HCs and patients with mTBI across three post‐injury time points (acute, 6‐month, and 12‐month). Age, sex, and education were included as covariates. Asterisks indicate significance levels: * *p* < 0.05, **p < 0.01, and ***p < 0.001.

Table 2 and Figure 2 summarize the TBI‐QoL results, in which higher TBI‐QoL scores indicate better quality of life. Cross‐sectional analyses revealed that patients with mTBI reported significantly poorer Physical Functioning (*p* < 0.001), more severe Physical Symptoms (*p* = 0.017), and reduced Social Participation (*p* < 0.001) than HCs during the acute phase. The remaining domains (Cognition, Emotion Well‐being, and Emotion Symptoms) did not differ significantly between groups at this time point. By the 6‐month post‐injury, significant differences remained only for Physical Functioning and Social Participation (both *p* = 0.043). By the 12‐month follow‐up, no significant differences were observed between the mTBI and HC groups for any TBI‐QoL domain (all *p* > 0.05).

**TABLE 2:** TBI‐QoL domain scores and statistical group comparisons between HCs and patients with mTBI.

Panel A. TBI‐QoL domain scores (Mean ± SD)				
Domain	HCs (*N* = 21)	Acute mTBI (*N* = 45)	6‐month mTBI (*N* = 33)	12‐month mTBI (*N* = 31)
Cognition	99.33 ± 37.86	79.18 ± 46.01	88.24 ± 45.74	79.16 ± 46.94
Emotion well‐being	58.29 ± 33.70	46.18 ± 21.54	51.67 ± 25.86	47.13 ± 16.82
Physical functioning	99.48 ± 25.38	60.71 ± 33.61	89.21 ± 28.97	87.45 ± 32.72
Social participation	116.62 ± 38.12	55.16 ± 34.65	91.67 ± 45.50	91.61 ± 48.16
Emotion symptoms	52.38 ± 5.61	56.93 ± 11.17	52.39 ± 6.93	54.58 ± 9.26
Physical symptoms	29.00 ± 4.09	37.40 ± 15.88	31.58 ± 5.96	32.74 ± 7.18

Panel B. FDR‐adjusted pairwise comparisons (*p*)						
Domain	HCs vs. Acute mTBI	HCs vs. 6‐month mTBI	HCs vs. 12‐month mTBI	Acute vs. 6‐month mTBI	6‐month vs. 12‐month mTBI	Acute vs. 12‐month mTBI
Cognition	0.068	0.249	0.059	0.307	0.985	0.721
Emotion well‐being	0.068	0.402	0.112	0.294	0.985	0.52
Physical functioning	< 0.001***	0.043*	0.059	< 0.001***	0.985	< 0.001***
Social participation	< 0.001***	0.043*	0.059	< 0.001***	0.997	< 0.001***
Emotion symptoms	0.094	0.942	0.463	0.056	0.985	0.349
Physical symptoms	**0.017**	0.178	0.059	0.056	0.985	0.149

*Note:* Values in Panel A are presented as mean ± SD. Panel B presents FDR‐adjusted *p*‐values for pairwise comparisons. Bold values indicate statistically significant differences after FDR correction (*p* < 0.05). Asterisks indicate FDR‐adjusted significance levels: * *p* < 0.05, ** *p* < 0.01, and *** *p* < 0.001.

Longitudinal analyses within the mTBI group demonstrated significant improvements in Physical Functioning and Social Participation between the acute visit and both the 6‐month and 12‐month follow‐up visits (all *p* < 0.001). No significant longitudinal changes were observed for any TBI‐QoL domain between the 6‐month and 12‐month follow‐up visits (all *p* ≥ 0985).

#### 3.2.2 NIH Toolbox Cognitive Tests

**FIGURE 3:**
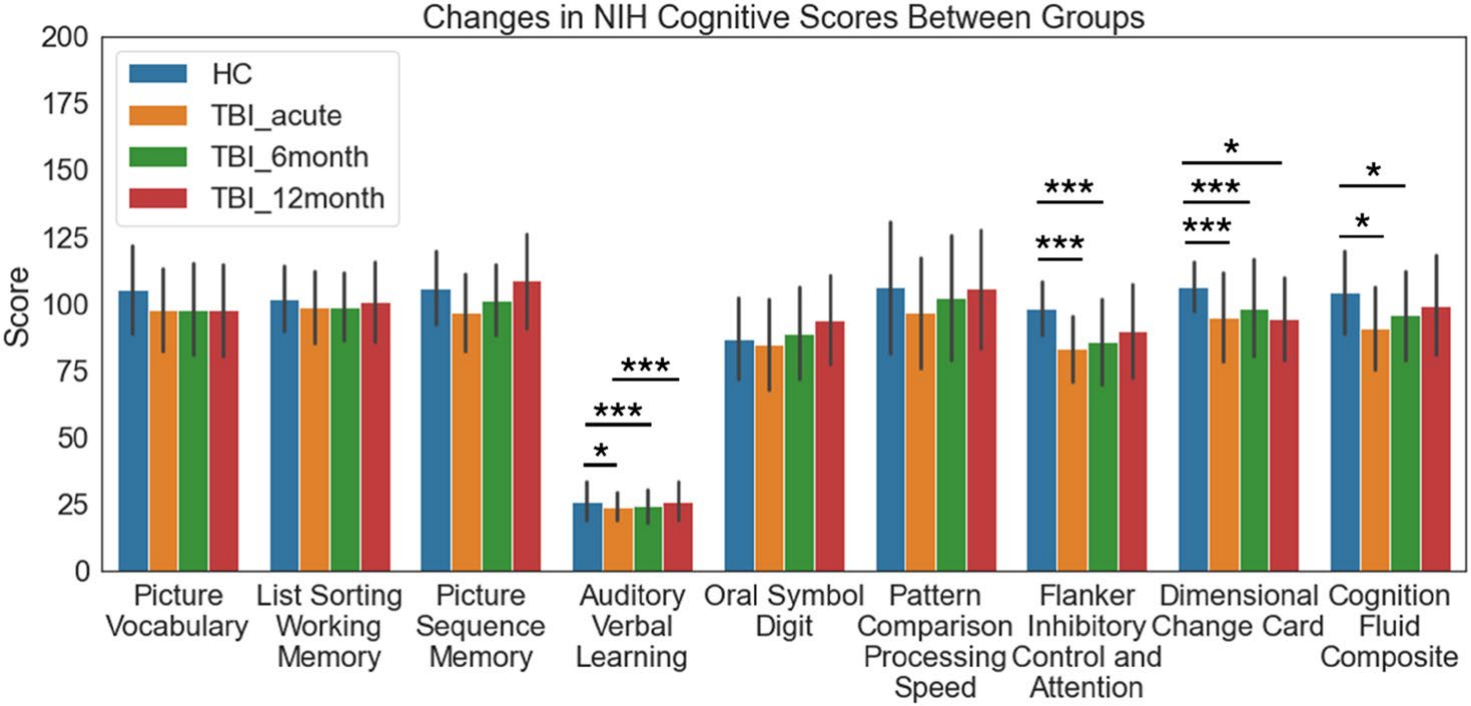
Linear mixed model (LMM) analysis results of NIH cognitive age‐adjusted scores by comparing HCs and patients with mTBI across three post‐injury time points (acute, 6‐month, and 12‐month). Age, sex, and education were included as covariates. Asterisks indicate significance levels: * *p* < 0.05, **p < 0.01, and ***p < 0.001.

Table 3 and Figure 3 summarize the NIH toolbox cognitive test results, in which higher scores indicate better cognitive performance. During the acute phase, patients with mTBI performed significantly worse than HCs on the auditory verbal learning test (*p* = 0.031), flanker inhibitory control & attention test (*p* < 0.001), dimensional change card sort test (*p* = 0.003), and the fluid cognition composite score (*p* = 0.003). At the 6‐month follow‐up, significant group differences persisted for the auditory verbal learning test, flanker inhibitory control & attention test, dimensional change card sort test, and the fluid cognition composite score (all *p* < 0.001). In addition, a significant difference was observed in the Picture Vocabulary Test (*p* = 0.012). By the 12‐month follow‐up, the only remaining significant difference was observed in the Dimensional Change Card Sort Test (*p* = 0.013).

**TABLE 3:** NIH toolbox cognition scores and statistical group comparisons between HCs and patients with mTBI.

Panel A. NIH toolbox cognition scores (Mean ± SD)				
NIH toolbox cognitive test	HCs (*N* = 21)	Acute mTBI (*N* = 46)	6‐month mTBI (*N* = 33)	12‐month mTBI (*N* = 31)
Picture vocabulary	104.86 ± 16.89	97.57 ± 15.87	97.70 ± 17.42	97.71 ± 17.95
List sorting working memory	105.62 ± 12.00	98.35 ± 13.63	98.73 ± 12.85	101.61 ± 15.00
Picture sequence memory	105.67 ± 14.00	96.57 ± 14.86	101.21 ± 13.48	109.42 ± 18.01
Auditory verbal learning	25.86 ± 7.41	23.98 ± 5.44	24.03 ± 6.36	26.23 ± 7.36
Oral symbol digit	106.81 ± 15.51	84.58 ± 16.98	88.87 ± 12.52	94.74 ± 16.24
Pattern comparison	105.81 ± 25.53	96.37 ± 21.07	102.03 ± 23.77	106.71 ± 22.38
Flanker inhibitory control & attention	97.95 ± 10.35	82.87 ± 12.46	85.86 ± 16.31	91.00 ± 17.03
Dimensional change card sort	106.19 ± 9.39	94.72 ± 16.92	98.15 ± 18.43	95.03 ± 14.75
Fluid cognition composite	103.90 ± 15.75	90.56 ± 15.79	95.33 ± 16.83	100.81 ± 17.67

Panel B. Pairwise comparisons (FDR‐adjusted *p*)						
NIH toolbox cognitive test	HCs vs. Acute mTBI	HCs vs. 6‐month mTBI	HCs vs. 12‐month mTBI	Acute vs. 6‐month mTBI	6‐month vs. 12‐month mTBI	Acute vs. 12‐month mTBI
Picture vocabulary	0.246	0.012	0.468	0.962	0.876	0.949
List sorting working memory	0.705	0.638	0.221	0.525	0.574	0.057
Picture sequence memory	0.439	0.192	0.993	0.843	0.675	0.521
Auditory verbal learning	0.031*	< 0.001***	0.795	0.525	0.574	< 0.001***
Oral symbol digit	< 0.001***	0.192	0.927	0.962	0.574	0.359
Pattern comparison	0.246	0.262	0.993	0.525	0.675	0.125
Flanker inhibitory control & attention	0.001***	< 0.001***	0.101	0.525	0.574	0.058
Dimensional change card Sort	0.003**	< 0.001***	**0.013**	0.525	0.574	0.982
Fluid cognition composite	0.003**	< 0.001***	0.604	0.525	0.574	0.057

*Note:* Values in Panel A are presented as mean ± SD. Panel B presents FDR‐adjusted *p*‐values for pairwise comparisons. Bold values indicate statistically significant differences after FDR correction (*p* < 0.05). Asterisks indicate FDR‐adjusted significance levels: * *p* < 0.05, ** *p* < 0.01, and *** *p* < 0.001.

Longitudinal analyses within the mTBI group demonstrated a significant improvement only in Auditory Verbal Learning Test scores between the acute visit to the 12‐month follow‐up (*p* < 0.001). No other NIH Toolbox Cognitive test or the Fluid Cognition Composite Score showed significant longitudinal changes across any of the follow‐up intervals.

### 3.3 Group‐Level Regional BEN Comparisons

All reported significant clusters survived a voxel‐wise threshold of *p* < 0.005 (uncorrected) and cluster‐level FDR correction (*p* < 0.05).

#### 3.3.1 Cross‐Sectional Analysis Results

**FIGURE 4:**
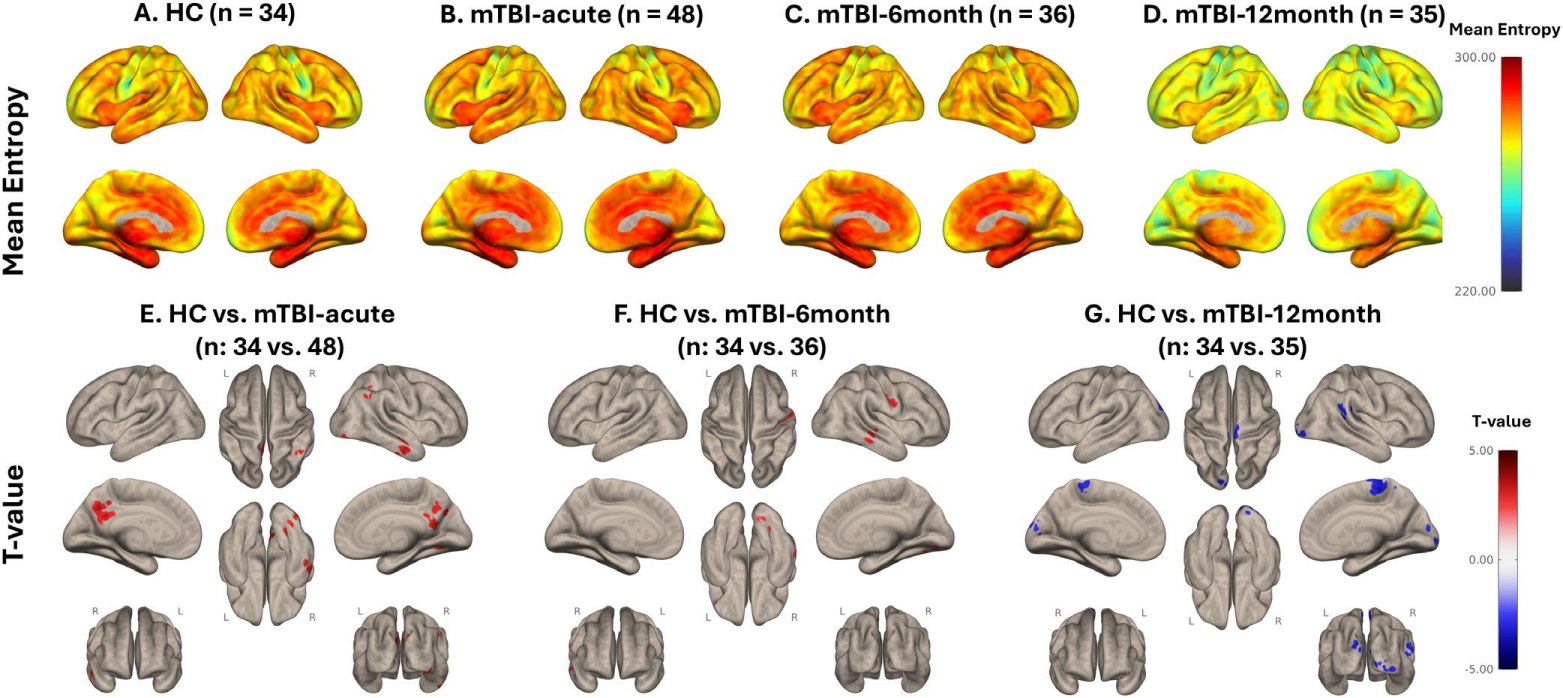
Brain entropy maps and voxel‐wise group comparisons between healthy controls (HCs) and patients with mTBI at three post‐injury time points. (A–D) Group‐averaged brain entropy maps for HCs and patients with mTBI at the acute, 6‐month, and 12‐month post‐injury visits, respectively. (E–G) Statistical *T*‐maps showing voxel‐wise group differences in brain entropy between HCs and patients with mTBI at the acute (E), 6‐month (F), and 12‐month (G) post‐injury visits. Warm colors indicate significantly higher brain entropy in patients with mTBI than in HCs, whereas cool colors indicate significantly lower brain entropy in patients with mTBI than in HCs. Group comparisons were performed using a voxel‐wise general linear model (GLM) with age and sex included as covariates. Statistical significance was defined as a voxel‐level threshold of *p* < 0.005 (uncorrected) and a cluster‐level false discovery rate (FDR)‐corrected *p* < 0.05.

Compared with HCs, patients with mTBI exhibited distinct patterns of regional BEN across the acute, 6‐month, and 12‐month post‐injury time points (Table 4 and Figure 4).

**TABLE 4:** Group differences in brain entropy between HC and mTBI at acute, 6‐month, and 12‐month post‐injury points.

Comparison	Direction of group difference	Peak MNI coordinate (x, y, z)	Cluster size (voxels)	Peak T	Cluster‐level FDR‐corrected *p*	Brain region(s)	Predominant functional network
HC vs. acute mTBI	mTBI>HC	(−8, −50, 26)	510	4.53	< 0.001***	Precuneus and posterior cingulate cortex	Default mode network (DMN)
		(58, −6, −28)	121	4.84	< 0.001***	Right anterior middle temporal gyrus, posterior middle temporal gyrus, and posterior inferior temporal gyrus	Temporal association network
		(30, −66, −4)	72	3.69	0.01*	Right occipital fusiform gyrus and inferior lateral occipital cortex	Visual network
		(44, −52, 44)	68	3.49	0.011*	Right angular gyrus	Frontoparietal network
HC vs. 6‐month mTBI	mTBI>HC	(16, −80, −6)	90	3.86	0.036*	Right occipital fusiform gyrus and lingual gyrus	Visual network
		(54, 0, 26)	86	3.64	0.036*	Right precentral gyrus	Sensorimotor network
		(50, −26, −6)	77	3.93	0.045*	Right posterior middle temporal gyrus	Temporal association network
HC vs. 12‐month mTBI	HC>mTBI	(4, −30, 60)	235	4.57	0.001**	Right precentral gyrus	Sensorimotor network
		(20, −94, 2)	152	4.3	0.009**	Right occipital pole	Visual network
		(−10, −88, 30)	113	3.83	0.036*	Left cuneal cortex and occipital pole	Visual network
		(46, −36, 24)	102	4.04	0.047*	Right posterior supramarginal gyrus	Frontoparietal network

*Note:* Peak MNI coordinates, cluster sizes, peak T‐values, and cluster‐level FDR‐corrected *p*‐values were obtained from SPM12. Anatomical labels were assigned using the Harvard–Oxford atlas implemented in the CONN toolbox. Bold values indicate statistically significant differences after FDR correction (*p* < 0.05). Asterisks indicate FDR‐corrected significance levels: * *p* < 0.05, ** *p* < 0.01, and *** *p* < 0.001.

In the acute phase, patients with mTBI exhibited significantly higher BEN than HCs in the precuneus/posterior cingulate cortex, right anterior and posterior middle temporal gyri, posterior inferior temporal gyrus, right occipital fusiform gyrus/inferior lateral occipital cortex, and right angular gyrus (Table 4). These clusters were predominantly located within the default mode, temporal association, visual, and frontoparietal networks.

At the 6‐month follow‐up, significantly higher BEN relative to HCs persisted in the right occipital fusiform gyrus/lingual gyrus, right precentral gyrus, and right posterior middle temporal gyrus (Table 4), predominantly located within the visual, sensorimotor, and temporal association networks.

By the 12‐month follow‐up, the direction of the group difference reversed, with patients with mTBI exhibiting significantly lower BEN than HCs in the right precentral gyrus, right occipital pole, left cuneal cortex/occipital pole, and right posterior supramarginal gyrus (Table 4). These clusters were predominantly located within the sensorimotor, visual, and frontoparietal networks.

#### 3.3.2 Longitudinal Analysis of Voxel‐Wise BEN Maps

**FIGURE 5:**
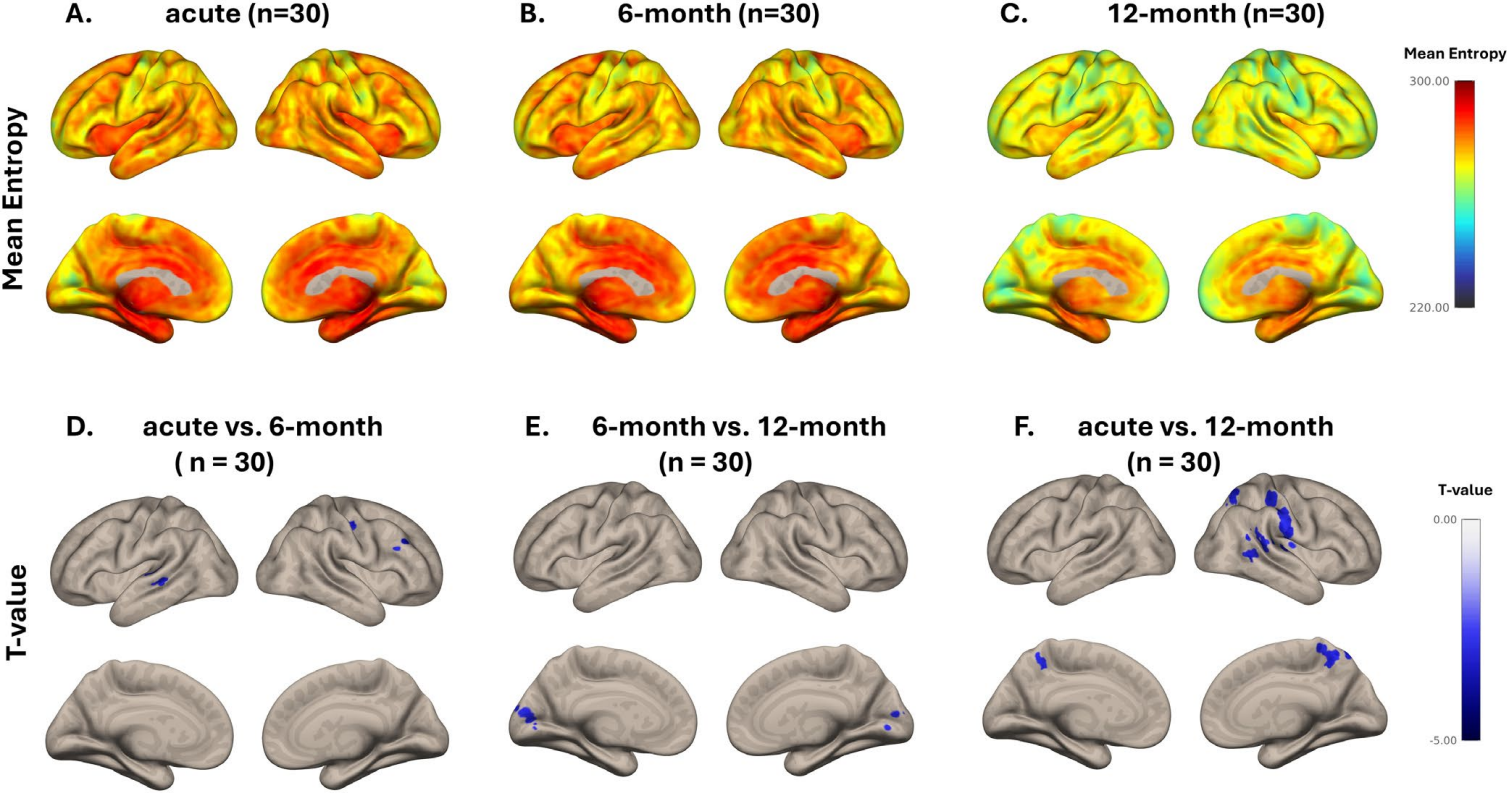
Brain entropy maps and longitudinal changes in brain entropy within patients with mTBI (*N* = 30) across the first year after injury. (A–C) Group‐averaged brain entropy maps for patients with mTBI at the acute, 6‐month, and 12‐month post‐injury visits, respectively. (D–F) Statistical T‐maps showing longitudinal changes in brain entropy between the acute and 6‐month visits (D), the 6‐month and 12‐month visits (E), and the acute and 12‐month visits (F). Warm colors indicate regions with significantly higher brain entropy at the later time point, whereas cool colors indicate regions with significantly lower brain entropy at the later time point. Longitudinal changes were assessed using voxel‐wise paired *t*‐tests. Statistical significance was defined as a voxel‐level threshold of *p* < 0.005 (uncorrected) and a cluster‐level false discovery rate (FDR)‐corrected *p* < 0.05.

Longitudinal changes in BEN were assessed in patients with mTBI who completed MRI at all three post‐injury time points (Table 5 and Figure 5).

**TABLE 5:** Longitudinal changes in brain entropy within the mTBI group.

Comparison	Direction of change	Peak MNI coordinate (x, y, z)	Cluster size (voxels)	Peak T	Cluster‐level FDR‐corrected *p*	Brain region(s)	Predominant functional network
Acute vs. 6‐month	6‐month<Acute	(−52, −32, 6)	62	4.13	0.006**	Left planum temporal, posterior superior temporal gyrus	Auditory/Language network
		(46, 30, 30)	53	5.34	0.01*	Right middle frontal gyrus	Frontoparietal/Executive control network
		(36, −12, 46)	49	3.99	0.011*	Right precentral gyrus and postcentral gyrus	Sensorimotor network
6‐month vs. 12‐month	12‐month < 6‐month	(−6, −88, 12)	167	4.6	0.034*	Left intra‐calcarine cortex, supra‐calcarine cortex, cuneal cortex	Visual network
Acute vs. 12‐month	12‐month<Acute	(6, −50, 58)	290	4.14	< 0.001***	Precuneus	Default mode network
		(60, −20, 32)	210	4.31	0.003**	Right posterior supramarginal gyrus, central opercular cortex	Frontoparietal network
		(62, −48, 4)	169	4.21	0.008**	Right lateral occipital cortex	Visual network
		(38, −28, 50)	165	4.3	0.008**	Right postcentral gyrus	Sensorimotor network

*Note:* Peak MNI coordinates, cluster sizes, peak T‐values, and cluster‐level FDR‐corrected *p*‐values were obtained from SPM12. Anatomical labels were assigned using the Harvard–Oxford atlas implemented in the CONN toolbox. Bold values indicate statistically significant differences after FDR correction (*p* < 0.05). Asterisks indicate FDR‐corrected significance levels: * *p* < 0.05, ** *p* < 0.01, and *** *p* < 0.001.

Between the acute phase and 6‐month follow‐up, BEN decreased significantly in the right middle frontal gyrus, left planum temporal/posterior superior temporal gyrus, and right precentral/postcentral gyri. These clusters were predominantly located within the frontoparietal, auditory/language, and sensorimotor networks.

Between 6 and 12 months post‐injury, a further decrease in BEN was observed in the bilateral intra‐calcarine and supra‐calcarine cortices and the left cuneal cortex, predominantly located in the visual network.

From the acute visit to the 12‐month follow‐up, BEN decreased significantly in the right posterior supramarginal gyrus/central opercular cortex, right postcentral gyrus, right lateral occipital cortex, and precuneus. These clusters were predominantly located within the frontoparietal, sensorimotor, visual, and default mode networks. No significant increases in BEN were identified in any of the longitudinal comparisons.

#### 3.3.3 Correlation Between Entropy Changes and Cognitive Function Changes Over Time

**FIGURE 6:**
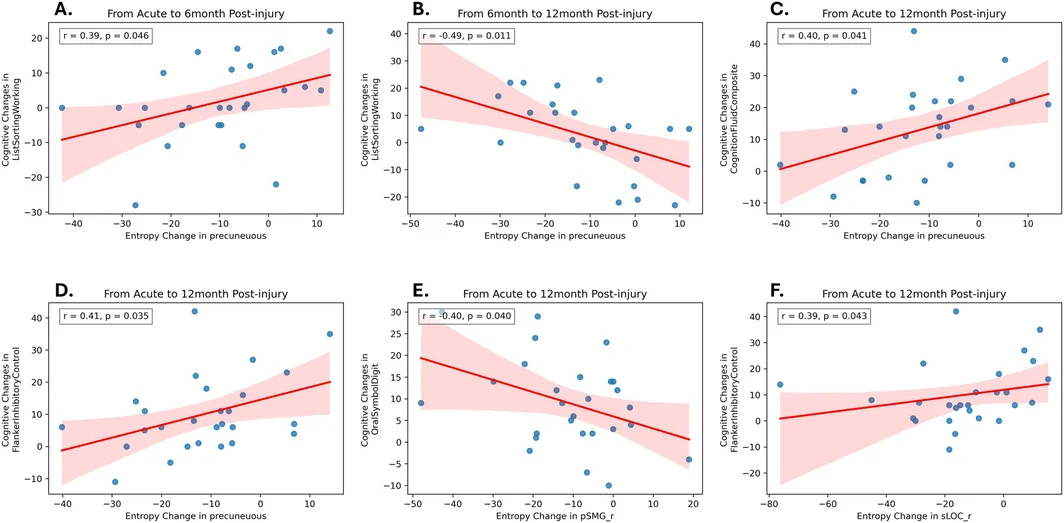
Associations between longitudinal changes in regional brain entropy and NIH Toolbox cognitive scores within the mTBI group. Scatter plots illustrate the associations identified using Spearman's rank correlation analysis for (A) acute → 6 months, (B) 6 months → 12 months, and (C–F) acute → 12 months. Only associations with uncorrected *p* < 0.05 are shown. None of the reported associations remained statistically significant after false discovery rate (FDR) correction.

Partial Pearson correlation analyses identified several nominally significant associations between longitudinal changes in regional BEN and changes in NIH Toolbox cognitive test scores. However, none remained statistically significant after false discovery rate (FDR) correction (*q* > 0.05) (Table 6 and Figure 6).

**TABLE 6:** Associations between longitudinal changes in regional BEN and changes in NIH Toolbox cognitive scores within the mTBI group.

Time interval	Brain region	NIH Toolbox Cognition test	Partial Pearson correlation	p‐unc	p‐FDR
6 months → 12 months	Precuneus	List Sorting Working Memory	−0.492	0.011	0.429
Acute → 12 months	Precuneus	Flanker Inhibitory Control and Attention	0.407	0.035	0.542
	Precuneus	Fluid Cognition Composite	0.396	0.041	0.542
	Right posterior supramarginal gyrus	Oral Symbol Digit	−0.397	0.04	0.542
	Right lateral occipital cortex	Flanker Inhibitory Control and Attention	0.392	0.043	0.542

During the interval from the acute visit to the 6‐month follow‐up, changes in brain entropy within the precuneus were positively correlated with changes in list sorting working memory test scores (ρ = 0.387, uncorrected *p* = 0.046, FDR‐corrected *q* = 0.379).

During the interval from the 6‐month to the 12‐month follow‐up, changes in BEN within the precuneus were negatively correlated with changes in List Sorting Working Memory Test scores (ρ = −0.492, uncorrected *p* = 0.011, FDR‐corrected *q* = 0.429).

Across the interval from the acute visit to the 12‐month follow‐up, changes in BEN within the precuneus were positively correlated with changes in Flanker Inhibitory Control and Attention Test scores (ρ = 0.407, uncorrected *p* = 0.035, FDR‐corrected *q* = 0.542) and Fluid Cognition Composite scores (ρ = 0.396, uncorrected *p* = 0.041, FDR‐corrected *q* = 0.542). In addition, changes in BEN within the right posterior supramarginal gyrus were negatively correlated with changes in Oral Symbol Digit Test scores (ρ = −0.397, uncorrected *p* = 0.040, FDR‐corrected *q* = 0.542), whereas changes in BEN within the right lateral occipital cortex were positively correlated with changes in Flanker Inhibitory Control and Attention Test scores (ρ = 0.392, uncorrected *p* = 0.043, FDR‐corrected *q* = 0.542).

## 4 Discussion

This longitudinal study combined patient‐reported outcomes, cognitive assessments, and resting‐state BEN to characterize recovery during the first year after mTBI. Three findings are notable. First, physical functioning and social participation improved substantially over time, and most group differences in cognitive performance were no longer evident by 12 months, although significant longitudinal improvement within the mTBI group was observed only for verbal learning. Second, BEN showed a time‐dependent spatial pattern, with higher BEN than HCs across default mode, temporal association, visual, frontoparietal, and sensorimotor regions during the acute and 6‐month stages, followed by lower BEN in sensorimotor, visual, and frontoparietal regions at 12 months. Third, longitudinal analyses demonstrated regional BEN decreases across recovery. Together, these findings suggest that mTBI is accompanied by evolving changes in the temporal complexity of resting‐state BOLD activity rather than a static abnormality.

### 4.1 Recovery of Quality of Life

TBI‐QoL measures indicated improvement in selected aspects of patient‐reported functioning after mTBI. During the acute phase, patients reported poorer physical functioning, greater physical symptom burden, and reduced social participation compared to HCs. This pattern is broadly consistent with previous studies showing acute impairments in physical and social functioning following mTBI, reflecting the immediate consequences of injury and activity limitations (Tulsky and Kisala 2019; Tyner et al. 2020; Capo‐Lugo et al. 2019; Heinemann et al. 2020; Poritz et al. 2020). Longitudinal analyses further demonstrated significant improvements in physical functioning and social participation by 6 months post‐injury, such that no significant group differences remained across any TBI‐QoL domain at 12 months. This pattern of recovery is consistent with previous studies showing that many individuals with mTBI experience substantial functional recovery within the first year after injury (Carroll et al. 2014; Elliott et al. 2023; Losoi et al. 2016; Stulemeijer et al. 2008).

### 4.2 Recovery of Cognitive Function

The NIH Toolbox Cognition Battery results indicated progressive recovery in objective cognitive performance following mTBI. During the acute phase, patients with mTBI exhibited deficits in episodic memory (Auditory Verbal Learning Test), executive function (Flanker Inhibitory Control & Attention Test and Dimensional Change Card Sort Test), and overall fluid cognition, as reflected by the Fluid Cognition Composite Score, consistent with previous reports of acute cognitive impairment following mTBI (Anderson 2021; de Freitas Cardoso et al. 2019; Nelson et al. 2019; Fraser et al. 2019; Coyle et al. 2023). Although some of these deficits persisted or new deficits emerged at 6 months, most cognitive deficits had resolved by 12 months post‐injury (de Freitas Cardoso et al. 2019; Rabinowitz and Levin 2014; Wylie et al. 2015). This pattern of recovery is consistent with previous studies demonstrating substantial cognitive improvement within the first year following mTBI (Nelson et al. 2023; Dery et al. 2025). However, executive function, as assessed by the Dimensional Change Card Sort Test, remained impaired at 12 months relative to HCs. This finding suggests that cognitive flexibility and set‐shifting may represent more vulnerable or slower‐recovering aspects of executive function following mTBI and warrant further investigation. Although episodic memory, as assessed by the Auditory Verbal Learning Test, showed significant longitudinal improvement within the mTBI group, the absence of significant within‐group changes in other cognitive domains may reflect substantial inter‐individual variability in recovery or improvements that were too subtle to detect with the present sample size.

### 4.3 Dynamic Changes in BEN

The BEN analyses revealed dynamic regionally specific changes associated with mTBI recovery. Our cross‐sectional analyses demonstrated time‐dependent alterations in BEN. During the acute phase, increased entropy in key hubs of the default mode network (DMN) and temporal association regions may reflect acute neural perturbation characterized by increased signal variability (Wang et al. 2014). This early hyper‐variability may represent compensatory neural reorganization or increased neural noise following injury, consistent with proposed models of DMN brain network disruption (Sours et al. 2013; Sharp et al. 2011; Zhou et al. 2012; Bonnelle et al. 2011; Li, Sun, et al. 2016; Mayer et al. 2010). At 6 months, increased entropy persisted but shifted spatially. By 12 months post‐injury, reduced entropy relative to HCs was observed in sensorimotor and visual regions (Castellanos et al. 2010; Simos et al. 2023). This transition from early relative hyper‐variability to later relative hypo‐variability suggests dynamic neural adaptation during recovery and may reflect increasing greater neural efficiency or continued functional reorganization. Consistent with the cross‐sectional findings, the longitudinal analyses demonstrated a progressive decrease in BEN over time within the mTBI group, indicating evolving neural dynamics during recovery. This progressive “settling” of BEN may reflect a transition toward more stable neural dynamics, or a process of network consolidation (Hillary and Grafman 2017; Sophie Su et al. 2016).

### 4.4 Exploratory Brain–Behavior Associations

The exploratory correlation analyses suggested potential brain–behavior relationships between longitudinal changes in BEN and cognitive performance during recovery from mTBI. Although several nominally significant associations were observed, none remained statistically significant after FDR correction; therefore, these findings should be interpreted as hypothesis‐generating and require validation in larger, independent longitudinal studies. Changes in BEN in precuneus were associated with changes in executive function and working memory across different recovery intervals, consistent with the established role of the precuneus as a central hub of the default mode network (DMN), which supports higher‐order cognitive processes including attention, executive function, and working memory (Raichle 2015; Dadario and Sughrue 2023; Menon 2023). In addition, exploratory associations involving the right posterior supramarginal gyrus and right lateral occipital cortex suggested that frontoparietal and visual networks may also contribute to cognitive recovery following mTBI. The direction of the association between BEN in precuneus and working memory differed between the acute‐to‐6‐month and 6‐to‐12‐month intervals, potentially reflecting evolving functional reorganization during recovery.

### 4.5 Limitations

This study has several limitations. First, the sample size was relatively modest, particularly for the behavioral analyses in HCs, which may have reduced the statistical power to detect subtle cognitive differences, brain–behavior associations, and potential interaction effects involving diagnosis, age, or sex. Although PTSD was not an exclusion criterion, only a small proportion of participants exhibited elevated post‐traumatic stress symptoms, precluding an evaluation of its potential influence on longitudinal BEN changes. Second, although demographic differences between groups were considered in the statistical analyses where appropriate, residual confounding cannot be completely excluded, and differences in racial distribution between groups may limit the generalizability of the findings. Finally, the present findings were based on a single cohort without an independent validation sample. Future studies should recruit larger, more diverse cohorts with longer follow‐up periods to validate the present findings, investigate the influence of demographic and clinical factors on recovery, and characterize long‐term neurobehavioral trajectories beyond the first year after injury. Combining BEN with other neuroimaging modalities, such as resting‐state functional connectivity, diffusion MRI, structural MRI, and cerebral perfusion imaging, may further improve understanding of the neural mechanisms underlying recovery and support the development of imaging biomarkers and targeted interventions for mTBI.

## 5 Conclusion

This longitudinal study provided a comprehensive neurobehavioral characterization of recovery following mTBI during the first year after injury. Patients with mTBI demonstrated substantial improvements in objective cognitive performance and aspects of quality of life by 12 months post‐injury. These behavioral improvements were accompanied by dynamic, time‐dependent alterations in BEN, characterized by a transition from early hyper‐variability to later hypo‐variability relative to HCs. Exploratory analyses also suggested potential associations between longitudinal changes in regional BEN and cognitive recovery. Together, these findings suggest that brain entropy may serve as a promising imaging biomarker for tracking the evolving neural dynamics underlying recovery following mTBI.

## Author Contributions

**Li Jiang:** conceptualization, methodology, software, formal analysis, data curation, visualization, writing – original draft, writing – review and editing. **Ze Wang:** conceptualization, methodology, software, supervision, writing – review and editing. **Steven Roys:** investigation, resources, data curation, writing – review and editing. **Rosy Linda Njonkou Tchoquessi:** investigation, data curation, writing – review and editing. **Prashant Raghavan:** supervision, writing – review and editing. **Neeraj Badjatia:** supervision, writing – review and editing. **Rao P. Gullapalli:** conceptualization, funding acquisition. **Jiachen Zhuo:** conceptualization, methodology, supervision, funding acquisition, writing – review and editing. All authors reviewed the manuscript, approved the final version, and agree to be accountable for all aspects of the work.

## Funding

This work was supported by the National Institutes of Health, 5R01NS105503.

## Conflicts of Interest

The authors declare no conflicts of interest.

## Supporting information


**Table S1:** TBI‐QoL domains, item banks, and scoring direction.
**Table S2:** NIH toolbox cognition battery (NIHTB‐CB) tests and cognitive functions.
**Table S3:** Comparison of mean FD between HCs and patients with mTBI.
**Figure S1:** Mean frame displacement (FD) comparison between HCs and patients with mTBI at acute stage. No significant difference between groups.

**Data S1:** Supporting Information.

## Data Availability

The data that support the findings of this study are available from the corresponding author upon reasonable request. The data are not publicly available because they contain information that could compromise the privacy of research participants.

## References

[jnr70153-bib-0001] Anderson, J. F. I. 2021. “Cognitive Complaint and Objective Cognition During the Post‐Acute Period After Mild Traumatic Brain Injury in Pre‐Morbidly Healthy Adults.” Brain Injury 35, no. 1: 103–113. 33459065 10.1080/02699052.2020.1859613

[jnr70153-bib-0002] Ashburner, J. , and K. J. Friston . 2005. “Unified Segmentation.” NeuroImage 26, no. 3: 839–851. 15955494 10.1016/j.neuroimage.2005.02.018

[jnr70153-bib-0003] Bonnelle, V. , R. Leech , K. M. Kinnunen , et al. 2011. “Default Mode Network Connectivity Predicts Sustained Attention Deficits After Traumatic Brain Injury.” Journal of Neuroscience 31, no. 38: 13442–13451. 21940437 10.1523/JNEUROSCI.1163-11.2011 PMC6623308

[jnr70153-bib-0004] Camargo, A. , G. Del Mauro , and Z. Wang . 2024. “Task‐Induced Changes in Brain Entropy.” Journal of Neuroscience Research 102, no. 2: e25310. 38400553 10.1002/jnr.25310 PMC10947426

[jnr70153-bib-0005] Cancelliere, C. , L. Verville , J. L. Stubbs , et al. 2023. “Post‐Concussion Symptoms and Disability in Adults With Mild Traumatic Brain Injury: A Systematic Review and Meta‐Analysis.” Journal of Neurotrauma 40, no. 11–12: 1045–1059. 36472218 10.1089/neu.2022.0185

[jnr70153-bib-0006] Capo‐Lugo, C. E. , P. A. Kisala , A. J. Boulton , S. W. Choi , A. W. Heinemann , and D. S. Tulsky . 2019. “Measuring Self‐Reported Physical Function in Individuals With TBI: Development of the TBI‐QOL Mobility and Upper Extremity Item Banks and Short Forms.” Journal of Head Trauma Rehabilitation 34, no. 5: 340–352. 31498232 10.1097/HTR.0000000000000511

[jnr70153-bib-0007] Carroll, L. J. , J. D. Cassidy , C. Cancelliere , et al. 2014. “Systematic Review of the Prognosis After Mild Traumatic Brain Injury in Adults: Cognitive, Psychiatric, and Mortality Outcomes: Results of the International Collaboration on Mild Traumatic Brain Injury Prognosis.” Archives of Physical Medicine and Rehabilitation 95, no. 3 Suppl: S152–S173. 24581903 10.1016/j.apmr.2013.08.300

[jnr70153-bib-0008] Cassidy, J. D. , C. Cancelliere , L. J. Carroll , et al. 2014. “Systematic Review of Self‐Reported Prognosis in Adults After Mild Traumatic Brain Injury: Results of the International Collaboration on Mild Traumatic Brain Injury Prognosis.” Archives of Physical Medicine and Rehabilitation 95, no. 3 Suppl: S132–S151. 24581902 10.1016/j.apmr.2013.08.299

[jnr70153-bib-0009] Castellanos, N. P. , N. Paúl , V. E. Ordóñez , et al. 2010. “Reorganization of Functional Connectivity as a Correlate of Cognitive Recovery in Acquired Brain Injury.” Brain 133, no. Pt 8: 2365–2381. 20826433 10.1093/brain/awq174

[jnr70153-bib-0010] Chang, D. , D. Song , J. Zhang , Y. Shang , Q. Ge , and Z. Wang . 2018. “Caffeine Caused a Widespread Increase of Resting Brain Entropy.” Scientific Reports 8, no. 1: 2700. 29426918 10.1038/s41598-018-21008-6 PMC5807546

[jnr70153-bib-0011] Coyle, H. L. , N. W. Bailey , J. Ponsford , and K. E. Hoy . 2023. “Recovery of Clinical, Cognitive and Cortical Activity Measures Following Mild Traumatic Brain Injury (mTBI): A Longitudinal Investigation.” Cortex 165: 14–25. 37245405 10.1016/j.cortex.2023.04.009

[jnr70153-bib-0012] Dadario, N. B. , and M. E. Sughrue . 2023. “The Functional Role of the Precuneus.” Brain 146, no. 9: 3598–3607. 37254740 10.1093/brain/awad181

[jnr70153-bib-0013] de Freitas Cardoso, M. G. , R. M. Faleiro , J. J. de Paula , et al. 2019. “Cognitive Impairment Following Acute Mild Traumatic Brain Injury.” Frontiers in Neurology 10: 198. 30906278 10.3389/fneur.2019.00198 PMC6418036

[jnr70153-bib-0014] Del Mauro, G. , Y. Li , J. Yu , et al. 2025. “Chronic Pain Is Associated With Greater Brain Entropy in the Prefrontal Cortex.” Journal of Pain 32: 105421. 40316037 10.1016/j.jpain.2025.105421 PMC12414511

[jnr70153-bib-0015] Del Mauro, G. , Y. Li , J. Yu , et al. 2026. “From Sensorimotor to Transmodal Cortex: Sleep Quality Aligns Brain Entropy With the Cortical Functional Gradient.” Sleep: zsag205. 10.1093/sleep/zsag205. 42518224

[jnr70153-bib-0016] Del Mauro, G. , L. S. Sevel , J. Boissoneault , and Z. Wang . 2024. “Divergent Association Between Pain Intensity and Resting‐State fMRI‐Based Brain Entropy in Different Age Groups.” Journal of Neuroscience Research 102, no. 5: e25341. 38751218 10.1002/jnr.25341 PMC11154588

[jnr70153-bib-0017] Del Mauro, G. , and Z. Wang . 2024. “Associations of Brain Entropy Estimated by Resting State fMRI With Physiological Indices, Body Mass Index, and Cognition.” Journal of Magnetic Resonance Imaging 59, no. 5: 1697–1707. 37578314 10.1002/jmri.28948 PMC10864678

[jnr70153-bib-0018] Del Mauro, G. , X. Zeng , and Z. Wang . 2025. “Normative Brain Entropy Across the Lifespan.” bioRxiv [Preprint]: 2025.05.08.652915. 10.1101/2025.05.08.652915.

[jnr70153-bib-0019] Dery, V. , G. Lafond , R. Picard , and P. Langevin . 2025. “Recovery From Mild Traumatic Brain Injury in the Nonathletic Population: A Systematic Review.” Neurotrauma Reports 6, no. 1: 355–374. 40697806 10.1089/neur.2025.0006 PMC12281108

[jnr70153-bib-0020] Dogra, S. , S. Arabshahi , J. Wei , et al. 2024. “Functional Connectivity Changes on Resting‐State fMRI After Mild Traumatic Brain Injury: A Systematic Review.” AJNR. American Journal of Neuroradiology 45, no. 6: 795–801. 38637022 10.3174/ajnr.A8204 PMC11288594

[jnr70153-bib-0021] Donghui Song, D. C. , J. Zhang , Q. Ge , Y.‐F. Zang , and Z. Wang . 2019. “Associations of Brain Entropy (BEN) to Cerebral Blood Flow and Fractional Amplitude of Low‐Frequency Fluctuations in the Resting Brain.” Brain Imaging and Behavior 13, no. 5: 486–1495. 10.1007/s11682-018-9963-4 30209786

[jnr70153-bib-0022] Donovan, J. , C. Cancelliere , and J. D. Cassidy . 2014. “Summary of the Findings of the International Collaboration on Mild Traumatic Brain Injury Prognosis.” Chiropractic & Manual Therapies 22, no. 1: 38. 25379171 10.1186/s12998-014-0038-3 PMC4221725

[jnr70153-bib-0023] Drachman, D. A. 2006. “Aging of the Brain, Entropy, and Alzheimer Disease.” Neurology 67, no. 8: 1340–1352. 17060558 10.1212/01.wnl.0000240127.89601.83

[jnr70153-bib-0024] Elliott, J. M. , D. M. Walton , S. R. Albin , et al. 2023. “Biopsychosocial Sequelae and Recovery Trajectories From Whiplash Injury Following a Motor Vehicle Collision.” Spine Journal 23, no. 7: 1028–1036. 10.1016/j.spinee.2023.03.005 PMC10330498 36958668

[jnr70153-bib-0025] Fraser, E. E. , M. G. Downing , K. Biernacki , D. P. McKenzie , and J. L. Ponsford . 2019. “Cognitive Reserve and Age Predict Cognitive Recovery After Mild to Severe Traumatic Brain Injury.” Journal of Neurotrauma 36, no. 19: 2753–2761. 31017049 10.1089/neu.2019.6430

[jnr70153-bib-0026] Gershon, R. C. , M. V. Wagster , H. C. Hendrie , N. A. Fox , K. F. Cook , and C. J. Nowinski . 2013. “NIH Toolbox for Assessment of Neurological and Behavioral Function.” Neurology 80, no. 11 Suppl 3: S2–S6. 23479538 10.1212/WNL.0b013e3182872e5f PMC3662335

[jnr70153-bib-0027] Heinemann, A. W. , P. A. Kisala , A. J. Boulton , et al. 2020. “Development and Calibration of the TBI‐QOL Ability to Participate in Social Roles and Activities and TBI‐QOL Satisfaction With Social Roles and Activities Item Banks and Short Forms.” Archives of Physical Medicine and Rehabilitation 101, no. 1: 20–32. 31473208 10.1016/j.apmr.2019.07.015

[jnr70153-bib-0028] Hillary, F. G. , and J. H. Grafman . 2017. “Injured Brains and Adaptive Networks: The Benefits and Costs of Hyperconnectivity.” Trends in Cognitive Sciences 21, no. 5: 385–401. 28372878 10.1016/j.tics.2017.03.003 PMC6664441

[jnr70153-bib-0029] Holdnack, J. A. , G. L. Iverson , N. D. Silverberg , D. S. Tulsky , and A. W. Heinemann . 2017. “NIH Toolbox Cognition Tests Following Traumatic Brain Injury: Frequency of Low Scores.” Rehabilitation Psychology 62, no. 4: 474–484. 29265868 10.1037/rep0000145 PMC5745050

[jnr70153-bib-0030] Jiang, W. , L. Cai , and Z. Wang . 2023. “Common Hyper‐Entropy Patterns Identified in Nicotine Smoking, Marijuana Use, and Alcohol Use Based on Uni‐Drug Dependence Cohorts.” Medical & Biological Engineering & Computing 61, no. 12: 3159–3166. 37718388 10.1007/s11517-023-02932-w PMC10842973

[jnr70153-bib-0031] Jordan, T. , M. R. Apostol , J. Nomi , and N. Petersen . 2024. “Unraveling Neural Complexity: Exploring Brain Entropy to Yield Mechanistic Insight in Neuromodulation Therapies for Tobacco Use Disorder.” Imaging Neuroscience 2: 1–17. 10.1162/imag_a_00061 PMC12224431 40800289

[jnr70153-bib-0032] Li, L. , G. Sun , K. Liu , et al. 2016. “White Matter Changes in Posttraumatic Stress Disorder Following Mild Traumatic Brain Injury: A Prospective Longitudinal Diffusion Tensor Imaging Study.” Chinese Medical Journal 129, no. 9: 1091–1099. 27098796 10.4103/0366-6999.180518 PMC4852678

[jnr70153-bib-0033] Li, Z. , Z. Fang , N. Hager , H. Rao , and Z. Wang . 2016. “Hyper‐Resting Brain Entropy Within Chronic Smokers and Its Moderation by Sex.” Scientific Reports 6: 29435. 27377552 10.1038/srep29435 PMC4932513

[jnr70153-bib-0034] Lin, L. , D. Chang , D. Song , Y. Li , and Z. Wang . 2022. “Lower Resting Brain Entropy Is Associated With Stronger Task Activation and Deactivation.” NeuroImage 249: 118875. 34998971 10.1016/j.neuroimage.2022.118875 PMC8881863

[jnr70153-bib-0035] Liu, P. , D. Song , X. Deng , et al. 2025. “The Effects of Intermittent Theta Burst Stimulation (iTBS) on Resting‐State Brain Entropy (BEN).” Neurotherapeutics 22, no. 3: e00556. 40050146 10.1016/j.neurot.2025.e00556 PMC12047393

[jnr70153-bib-0036] Liu, X. , D. Song , Y. Yin , et al. 2020. “Altered Brain Entropy as a Predictor of Antidepressant Response in Major Depressive Disorder.” Journal of Affective Disorders 260: 716–721. 31563070 10.1016/j.jad.2019.09.067

[jnr70153-bib-0037] Losoi, H. , N. D. Silverberg , M. Wäljas , et al. 2016. “Recovery From Mild Traumatic Brain Injury in Previously Healthy Adults.” Journal of Neurotrauma 33, no. 8: 766–776. 26437675 10.1089/neu.2015.4070

[jnr70153-bib-0038] Mayer, A. R. , J. Ling , M. V. Mannell , et al. 2010. “A Prospective Diffusion Tensor Imaging Study in Mild Traumatic Brain Injury.” Neurology 74, no. 8: 643–650. 20089939 10.1212/WNL.0b013e3181d0ccdd PMC2830922

[jnr70153-bib-0039] McCrea, M. A. , J. T. Giacino , J. Barber , et al. 2021. “Functional Outcomes Over the First Year After Moderate to Severe Traumatic Brain Injury in the Prospective, Longitudinal TRACK‐TBI Study.” JAMA Neurology 78, no. 8: 982–992. 34228047 10.1001/jamaneurol.2021.2043 PMC8261688

[jnr70153-bib-0040] McGeown, J. P. , M. Pedersen , R. Mito , et al. 2026. “Neuroimaging Correlates of Symptom Burden and Functional Recovery Following Mild Traumatic Brain Injury: A Systematic Review.” NeuroImage: Clinical 49: 103910. 41496379 10.1016/j.nicl.2025.103910 PMC12811548

[jnr70153-bib-0041] McInnes, K. , C. L. Friesen , D. E. MacKenzie , D. A. Westwood , and S. G. Boe . 2017. “Mild Traumatic Brain Injury (mTBI) and Chronic Cognitive Impairment: A Scoping Review.” PLoS One 12, no. 4: e0174847. 28399158 10.1371/journal.pone.0174847 PMC5388340

[jnr70153-bib-0042] Menon, V. 2023. “20 Years of the Default Mode Network: A Review and Synthesis.” Neuron 111, no. 16: 2469–2487. 37167968 10.1016/j.neuron.2023.04.023 PMC10524518

[jnr70153-bib-0043] Morfini, F. , S. Whitfield‐Gabrieli , and A. Nieto‐Castanon . 2023. “Functional Connectivity MRI Quality Control Procedures in CONN.” Frontiers in Neuroscience 17: 1092125. 37034165 10.3389/fnins.2023.1092125 PMC10076563

[jnr70153-bib-0044] Mott, T. F. , M. L. McConnon , and B. P. Rieger . 2012. “Subacute to Chronic Mild Traumatic Brain Injury.” American Family Physician 86, no. 11: 1045–1051. 23198672

[jnr70153-bib-0045] Nelson, L. D. , N. R. Temkin , J. Barber , et al. 2023. “Functional Recovery, Symptoms, and Quality of Life 1 to 5 Years After Traumatic Brain Injury.” JAMA Network Open 6, no. 3: e233660. 36939699 10.1001/jamanetworkopen.2023.3660 PMC10028488

[jnr70153-bib-0046] Nelson, L. D. , N. R. Temkin , S. Dikmen , et al. 2019. “Recovery After Mild Traumatic Brain Injury in Patients Presenting to US Level I Trauma Centers: A Transforming Research and Clinical Knowledge in Traumatic Brain Injury (TRACK‐TBI) Study.” JAMA Neurology 76, no. 9: 1049–1059. 31157856 10.1001/jamaneurol.2019.1313 PMC6547159

[jnr70153-bib-0047] Poritz, J. M. P. , M. Sherer , P. A. Kisala , D. Tulsky , L. Leon‐Novelo , and E. Ngan . 2020. “Responsiveness of the Traumatic Brain Injury‐Quality of Life (TBI‐QOL) Measurement System.” Archives of Physical Medicine and Rehabilitation 101, no. 1: 54–61. 29407517 10.1016/j.apmr.2017.11.018

[jnr70153-bib-0048] Rabinowitz, A. R. , and H. S. Levin . 2014. “Cognitive Sequelae of Traumatic Brain Injury.” Psychiatric Clinics of North America 37, no. 1: 1–11. 24529420 10.1016/j.psc.2013.11.004 PMC3927143

[jnr70153-bib-0049] Raichle, M. E. 2015. “The Brain's Default Mode Network.” Annual Review of Neuroscience 38: 433–447. 10.1146/annurev-neuro-071013-014030 25938726

[jnr70153-bib-0050] Sharp, D. J. , C. F. Beckmann , R. Greenwood , et al. 2011. “Default Mode Network Functional and Structural Connectivity After Traumatic Brain Injury.” Brain 134, no. Pt 8: 2233–2247. 21841202 10.1093/brain/awr175

[jnr70153-bib-0051] Sheldrake, E. , H. al‐Hakeem , B. Lam , et al. 2022. “Mental Health Outcomes Across the Lifespan in Individuals With Persistent Post‐Concussion Symptoms: A Scoping Review.” Frontiers in Neurology 13: 850590. 35481264 10.3389/fneur.2022.850590 PMC9035995

[jnr70153-bib-0052] Simos, N. J. , K. Manolitsi , A. I. Luppi , et al. 2023. “Chronic Mild Traumatic Brain Injury: Aberrant Static and Dynamic Connectomic Features Identified Through Machine Learning Model Fusion.” Neuroinformatics 21, no. 2: 427–442. 36456762 10.1007/s12021-022-09615-1 PMC10085953

[jnr70153-bib-0053] Song, D. , D. Chang , J. Zhang , et al. 2019. “Reduced Brain Entropy by Repetitive Transcranial Magnetic Stimulation on the Left Dorsolateral Prefrontal Cortex in Healthy Young Adults.” Brain Imaging and Behavior 13, no. 2: 421–429. 29629499 10.1007/s11682-018-9866-4

[jnr70153-bib-0054] Song, D. , X. P. Deng , D. Chang , and Z. Wang . 2025. “Altered Resting‐State Brain Entropy by Repetitive Transcranial Magnetic Stimulation Across the Human Cortex.” Cerebral Cortex 35, no. 7: bhalf171. 10.1093/cercor/bhaf171. 40650633

[jnr70153-bib-0055] Song, D. , and Z. Wang . 2025a. “The Relationships of Resting‐State Brain Entropy (BEN), Ovarian Hormones and Behavioral Inhibition and Activation Systems (BIS/BAS).” NeuroImage 312: 121226. 40262490 10.1016/j.neuroimage.2025.121226

[jnr70153-bib-0056] Song, D. , and Z. Wang . 2025b. “Age‐Dependent Effects of Intranasal Oxytocin Administration Were Revealed by Resting Brain Entropy (BEN).” Behavioural Brain Research 500: 115985. 41360155 10.1016/j.bbr.2025.115985

[jnr70153-bib-0057] Sophie Su, Y. , A. Veeravagu , and G. Grant . 2016. “Neuroplasticity After Traumatic Brain Injury.” In Translational Research in Traumatic Brain Injury, edited by D. Laskowitz and G. Grant . CRC Press/Taylor & Francis. 26583189

[jnr70153-bib-0058] Sours, C. , J. Zhuo , J. Janowich , B. Aarabi , K. Shanmuganathan , and R. P. Gullapalli . 2013. “Default Mode Network Interference in Mild Traumatic Brain Injury ‐ a Pilot Resting State Study.” Brain Research 1537: 201–215. 23994210 10.1016/j.brainres.2013.08.034 PMC3835746

[jnr70153-bib-0059] Sours, C. , J. Zhuo , S. Roys , K. Shanmuganathan , and R. P. Gullapalli . 2015. “Disruptions in Resting State Functional Connectivity and Cerebral Blood Flow in Mild Traumatic Brain Injury Patients.” PLoS One 10, no. 8: e0134019. 26241476 10.1371/journal.pone.0134019 PMC4524606

[jnr70153-bib-0060] Stulemeijer, M. , S. van der Werf , G. F. Borm , and P. E. Vos . 2008. “Early Prediction of Favourable Recovery 6 Months After Mild Traumatic Brain Injury.” Journal of Neurology, Neurosurgery, and Psychiatry 79, no. 8: 936–942. 17951281 10.1136/jnnp.2007.131250

[jnr70153-bib-0061] Tulsky, D. S. , N. E. Carlozzi , J. Holdnack , et al. 2017. “Using the NIH Toolbox Cognition Battery (NIHTB‐CB) in Individuals With Traumatic Brain Injury.” Rehabilitation Psychology 62, no. 4: 413–424. 29265862 10.1037/rep0000174 PMC6462276

[jnr70153-bib-0062] Tulsky, D. S. , and P. A. Kisala . 2019. “An Overview of the Traumatic Brain Injury‐Quality of Life (TBI‐QOL) Measurement System.” Journal of Head Trauma Rehabilitation 34, no. 5: 281–288. 31498227 10.1097/HTR.0000000000000531

[jnr70153-bib-0063] Tyner, C. E. , A. J. Boulton , M. Sherer , P. A. Kisala , J. J. Glutting , and D. S. Tulsky . 2020. “Development of Composite Scores for the TBI‐QOL.” Archives of Physical Medicine and Rehabilitation 101, no. 1: 43–53. 31875840 10.1016/j.apmr.2018.05.036

[jnr70153-bib-0064] Vedaei, F. , A. B. Newberg , M. Alizadeh , et al. 2021. “Resting‐State Functional MRI Metrics in Patients With Chronic Mild Traumatic Brain Injury and Their Association With Clinical Cognitive Performance.” Frontiers in Human Neuroscience 15: 768485. 35027887 10.3389/fnhum.2021.768485 PMC8751629

[jnr70153-bib-0065] Wang, Z. 2021. “The Neurocognitive Correlates of Brain Entropy Estimated by Resting State fMRI.” NeuroImage 232: 117893. 33621695 10.1016/j.neuroimage.2021.117893 PMC8138544

[jnr70153-bib-0066] Wang, Z. , and Alzheimer's Disease Neuroimaging Initiative . 2020. “Brain Entropy Mapping in Healthy Aging and Alzheimer's Disease.” Frontiers in Aging Neuroscience 12: 596122. 33240080 10.3389/fnagi.2020.596122 PMC7683386

[jnr70153-bib-0067] Wang, Z. , Y. Li , A. R. Childress , and J. A. Detre . 2014. “Brain Entropy Mapping Using fMRI.” PLoS One 9, no. 3: e89948. 24657999 10.1371/journal.pone.0089948 PMC3962327

[jnr70153-bib-0068] Wang, Z. , J. Suh , D. Duan , et al. 2017. “A Hypo‐Status in Drug Dependent Brain Revealed by Multi‐Modal MRI.” Addiction Biology 22, no. 6: 1622–1631. 27654848 10.1111/adb.12459 PMC5362359

[jnr70153-bib-0069] Weintraub, S. , S. S. Dikmen , R. K. Heaton , et al. 2014. “The Cognition Battery of the NIH Toolbox for Assessment of Neurological and Behavioral Function: Validation in an Adult Sample.” Journal of the International Neuropsychological Society 20, no. 6: 567–578. 24959840 10.1017/S1355617714000320 PMC4103959

[jnr70153-bib-0070] Wylie, G. R. , K. Freeman , A. Thomas , et al. 2015. “Cognitive Improvement After Mild Traumatic Brain Injury Measured With Functional Neuroimaging During the Acute Period.” PLoS One 10, no. 5: e0126110. 25962067 10.1371/journal.pone.0126110 PMC4427352

[jnr70153-bib-0071] Xue, S. W. , Q. Yu , Y. Guo , D. Song , and Z. Wang . 2019. “Resting‐State Brain Entropy in Schizophrenia.” Comprehensive Psychiatry 89: 16–21. 30576960 10.1016/j.comppsych.2018.11.015

[jnr70153-bib-0072] Yin, N. , H. Wang , Z. Wang , K. Feng , G. Xu , and S. Yin . 2023. “A Study of Brain Networks Associated With Freezing of Gait in Parkinson's Disease Using Transfer Entropy Analysis.” Brain Research 1821: 148610. 37783260 10.1016/j.brainres.2023.148610

[jnr70153-bib-0073] Zhan, J. , L. Gao , F. Zhou , et al. 2015. “Decreased Regional Homogeneity in Patients With Acute Mild Traumatic Brain Injury: A Resting‐State fMRI Study.” Journal of Nervous and Mental Disease 203, no. 10: 786–791. 26348589 10.1097/NMD.0000000000000368

[jnr70153-bib-0074] Zhou, F. , Y. Zhuang , H. Gong , J. Zhan , M. Grossman , and Z. Wang . 2016. “Resting State Brain Entropy Alterations in Relapsing Remitting Multiple Sclerosis.” PLoS One 11, no. 1: e0146080. 26727514 10.1371/journal.pone.0146080 PMC4699711

[jnr70153-bib-0075] Zhou, Y. , M. P. Milham , Y. W. Lui , et al. 2012. “Default‐Mode Network Disruption in Mild Traumatic Brain Injury.” Radiology 265, no. 3: 882–892. 23175546 10.1148/radiol.12120748 PMC3504316

[jnr70153-bib-0076] Zhuo, J. , P. Raghavan , J. Li , et al. 2024. “Longitudinal Assessment of Glymphatic Changes Following Mild Traumatic Brain Injury: Insights From Perivascular Space Burden and DTI‐ALPS Imaging.” Frontiers in Neurology 15: 1443496. 39170078 10.3389/fneur.2024.1443496 PMC11335690

