# Supplementary material for: Resting‐State Brain Entropy Reveals Altered Neural Dynamics and Cognitive Associations in Mild Traumatic Brain Injury: A Longitudinal Study
Source: J Neurosci Res. 2026 Aug 28;104(9):e70153. doi: 10.1002/jnr.70153 (PMC13525400; doi:10.1002/jnr.70153)
Supplement: Supplementary file 2 [file JNR-104-e70153-s002.docx]

**Supplemental Table 1. TBI-QoL domains, item banks, and scoring direction.**

**Supplemental Table 2: NIH toolbox Cognition Battery (NIHTB-CB) Tests and Cognitive Functions**

**Supplemental Table 3. Comparison of mean FD between HCs and patients with mTBI.**

| **Group** | ***N*** | **Mean FD (mm), mean ± SD** |
| --- | --- | --- |
| HC | 34 | 0.194 ± 0.083 |
| mTBI | 48 | 0.209 ± 0.081 |
| Group comparison |  | Mann–Whitney U test, *p* = 0.300 |
| *Notes. FD = framewise displacement. No significant difference in mean FD was observed between the HC and mTBI groups (p = 0.300).* | | |

Supplemental Figure 1. Mean Frame Displacement (FD) comparison between HCs and patients with mTBI at acute stage. No significant difference between groups.
